# Carbapenem Resistance among Marine Bacteria—An Emerging Threat to the Global Health Sector

**DOI:** 10.3390/microorganisms9102147

**Published:** 2021-10-14

**Authors:** Dewa A.P. Rasmika Dewi, Torsten Thomas, Ana Masara Ahmad Mokhtar, Noreen Suliani Mat Nanyan, Siti Balqis Zulfigar, Nor Hawani Salikin

**Affiliations:** 1 School of Medicine, International University of Health and Welfare, Narita 286-8686, Japan; d-dewi@iuhw.ac.jp; 2 Faculty of Medicine and Health Sciences, Udayana University, Bali 80232, Indonesia; 3 Centre for Marine Science and Innovation, School of Biological, Earth and Environmental Sciences, The University of New South Wales, Sydney 2052, Australia; t.thomas@unsw.edu.au; 4 School of Industrial Technology, Universiti Sains Malaysia, Gelugor 11800, Penang, Malaysia; anamasara@usm.my (A.M.A.M.); noreen_nanyan@usm.my (N.S.M.N.); balqiszulfigar@usm.my (S.B.Z.)

**Keywords:** infectious diseases, safe water access, marine bacteria, carbapenem resistance

## Abstract

The emergence of antibiotic resistance among pathogenic microorganisms is a major issue for global public health, as it results in acute or chronic infections, debilitating diseases, and mortality. Of particular concern is the rapid and common spread of carbapenem resistance in healthcare settings. Carbapenems are a class of critical antibiotics reserved for treatment against multidrug-resistant microorganisms, and resistance to this antibiotic may result in limited treatment against infections. In addition to in clinical facilities, carbapenem resistance has also been identified in aquatic niches, including marine environments. Various carbapenem-resistant genes (CRGs) have been detected in different marine settings, with the majority of the genes incorporated in mobile genetic elements, i.e., transposons or plasmids, which may contribute to efficient genetic transfer. This review highlights the potential of the marine environment as a reservoir for carbapenem resistance and provides a general overview of CRG transmission among marine microbes.

## 1. Introduction

Antibiotic resistance (AR) is a public health crisis that leads to increasingly complex and expensive treatments, longer hospital stays, and higher mortality [1]. The Centre for Disease Prevention and Control (CDC) has estimated that in the United States alone, two million people are infected by antibiotic-resistant bacteria (ARB), resulting in 23,000 fatalities every year [2]. The WHO estimates that this number will increase to approximately 10 million deaths per year by 2050 if the AR problem is not addressed [3,4]. ARB can be found in humans, animals, plants, and the environment, such as in water and soil [5], and may spread among humans, between humans and animals, from humans or animals to the environment, or vice versa [6,7,8,9,10]. The factors contributing to the emergence and spread of ARB include the overuse or misuse of antimicrobials; poor infection prevention and control in healthcare facilities; lack of access to clean water, sanitation, and hygiene for humans and animals; lack of knowledge and awareness; and lack of legislation enforcement [5]. Furthermore, the rapid global spread of ARB that have acquired new resistance mechanisms has, alarmingly, resulted in multi- and pan-resistant bacteria, also known as “superbugs” [5]. Consequently, only a few antibiotics are left to treat human infections caused by these multi-drug-resistant bacteria [11,12,13]. The WHO, therefore, suggests that prevention and containment methods are required to decrease the spread of ARB, and these include improved methods for antibiotic prescription, the regulation of antibiotics use, the development of new antimicrobial drugs and vaccines, and improved surveillance of ARB [5].

The most diverse ARB worldwide are those that produce beta-lactamases (β-lactam hydrolysing enzymes). More than 1000 beta-lactamases, including newly discovered classes of genes and their mutations, have been identified [14]. The β-lactams belonging to the class of carbapenems are very efficient antibiotics that are widely used to treat severe or high-risk bacterial infections. Due to their broad spectrum of activity and efficacy against both Gram-positive and Gram-negative bacteria, carbapenems are considered a “last resort” antibiotic to treat severely ill patients or those suspected of carrying multi-resistant bacteria [15]. Alarmingly, carbapenem resistance (CR) has been reported globally [15,16,17], with the synthesis of carbapenemases being the most common mechanism underpinning resistance [15,17,18,19]. Furthermore, several cases of CR have been reported in bacteria outside the hospital setting, including rivers [20], sewage [21,22], and marine waters [23,24,25,26]. This review summarizes the knowledge on the spread of CR outside of the clinical setting with particular focus on the potential of the marine environment to act as a reservoir for the genetic transfer of CR among bacteria.

## 2. Mechanisms of Carbapenem Resistance

Compared to penicillins and cephalosporins, carbapenems have an overall broader antimicrobial spectrum [27]. Generally, carbapenems enter bacteria through porins, and after passing the periplasmic space, acylate penicillin-binding proteins (PBPs), which stops the formation of peptidoglycan as well as the cell wall, ultimately leading to cell lysis [28]. A key factor in the efficacy of carbapenems is their ability to bind to multiple different PBPs [15].

Structural changes in or mutations of PBPs, which typically occur in Gram-positive cocci, can contribute to CR. Alterations in affinity, the expression of efflux pumps, and the aberrant production of carbapenemases may also promote CR [15,17]. Several carbapenem-resistant bacteria (CRB), such as *Klebsiella pneumoniae*, *Acinetobacter baumannii,* and *Pseudomonas aeruginosa*, are known to possess a combination of these resistance mechanisms [15]. Additionally, alteration in porins or membrane protein functions can lead to reduced diffusion of carbapenem into the periplasm [29]. This has been observed in *K*. *pneumoniae*, which acquired CR through a lack of the outer membrane proteins OmpK35 and OmpK36 [30]. Mutations of the regulator gene *marR* and lack of OmpF and OmpC porins have also been correlated with CR in *Escherichia coli* strains from clinical samples [31].

Carbapenemases can hydrolyse almost all β-lactam antibiotics, including penicillins, cephalosporins, monobactams, and carbapenems [16]. The expression of carbapenemases seems to play an important role in the spread of CR [28,32]. The wide range of resistance phenotypes observed among carbapenemase-producing isolates is associated with the level of enzyme expression and other resistant mechanisms, such as the expression of other β-lactamases, efflux pumps, or altered permeability [17,33]. 

Carbapenemases are divided into two major types, serine carbapenemases and metallo-β-lactamases, which have serine and zinc ions at their active sites, respectively [16]. Serine carbapenemases are mostly chromosomally encoded [34,35,36], and metallo-β-lactamases are often found on plasmids [37,38,39], although recent studies have reported that this pattern has shifted, with both classes being plasmid-encoded [19,40,41,42]. The metallo-β-lactamases are characterized by their ability to hydrolyse extended-spectrum cephalosporins (cefotaxime, ceftazidime, and cefepime) [43]. The hydrolysis ability is determined by the interaction of zinc ions on their active site and the β-lactams [44]. New Delhi Metallo-β-lactamase (NDM) is the latest type of metallo-β-lactamase to be identified. NDM-1 is the major variant and is found mostly in *Enterobacteriaceae* [45]. It was first described in *K*. *pneumoniae* and *E*. *coli* isolates in 2008 in Sweden from an Indian patient transferred from a New Delhi hospital [45,46]. The *bla*_NDM-1_ gene can be carried by different plasmid types (IncA/C, IncF, and IncL/M), and in rare cases, is chromosomally integrated [45]. In addition, most plasmids with *bla*_NDM-1_ genes harbor a variety of other resistance genes, such as *bla*_TEM-1_, *bla*_OXA-1_, *bla*_OXA-10_, and *bla*_CMY_ (encoding various β-lactamases); *qnrA6* and *qnrB1* (encoding quinolone resistance); *arr-2* (encoding rifampicin resistance); *sul-2* (encoding sulphonamide resistance); *cmlA* (encoding chloramphenicol resistance); and *ereC* (encoding macrolide resistance) [45].

## 3. Epidemiology and Distribution of Carbapenem Resistance

The CDC reports that the highest mortality rate related to AR occurs in healthcare settings, including hospitals. Hospitals are one of a number of sources of organisms with multiple AR, so-called “superbugs”, which create a broad concern for public health. Carbapenemase-resistant *Enterobacteriaceae* have been highlighted as a source of life-threatening nosocomial infection [16], and the epidemiological status of CRB is progressively worsening. Giske et al. reported that in Europe, an outbreak of CR that occurred during the 2000s in several Mediterranean countries was caused mainly by carbapenemase-producing *P*. *aeruginosa* [47]. In addition, the OXA-48-like enzymes and NDM-producing *Enterobacteriaceae* have also spread rapidly in several European countries [47]. In the US, *Klebsiella pneumoniae* carbapenemase (KPC) is the predominant carbapenemase among *Enterobacteriaceae*, and the first KPC was isolated from a patient in North Carolina in 1996. Up to 2013, the CDC reported that at least one KPC-producing *Enterobacteriaceae* had been identified in 46 states and mostly found in *K*. *pneumoniae*, *E*. *coli,* and *Enterobacter* spp. [48]. In Australia, CRGs encoding KPC, imipenem-hydrolysing β-lactamase (IMP), NDM, or oxacillin-hydrolysing carbapenemase (OXA) have been identified in human pathogens. A plasmid-mediated *bla*_NDM-5_ was identified in an *E*. *coli* isolate from a urine sample of a patient in Brisbane [49]. Isolates belonging to species *Serratia marcescens*, *K*. *pneumoniae*, *P*. *aeruginosa*, *E*. *coli*, and *Enterobacter cloacae* and containing IMP-4 resistance genes were recovered from different patients hospitalized in Melbourne [50]. In addition, multiple CRGs (e.g., *bla*_IMP-4_ and *bla*_OXA-58_) were also found in an *Acinetobacter junii* isolate from a blood sample in Melbourne [51]. Furthermore, some carbapenemases were also isolated from *Enterobacteriaceae* species found in various animals and in healthy humans [52]. These carbapenemases included KPC, OXA NDM, VIM, OXA, IMP from *Citrobacter* spp., *Cronobacter sakazakii*, *Enterobacter* spp., *E*. *coli*, *Klebsiella oxytoca*, *K*. *pneumoniae*, *Morganella* spp., *Proteus* spp., *Providencia* spp., and *Salmonella* spp. [52,53,54,55].

Aside from human-populated environments, CR can also spread into the natural milieu, including aquatic environments [42,56,57,58]. CRGs, such as *bla*_NDM-1_, *bla*_KPC-2_, and *bla*_OXA-58_, have also been reported in known environmental bacteria, such as *Acinetobacter johnsonii* [59] and *Acinetobacter towneri* [60]. Imipenem-hydrolysing β-lactamase-2 has also been found in *Enterobacter asburiae* isolated from USA rivers [32]. A novel carbapenemase, BIC-2, was identified in water samples from the Seine River, Paris. This enzyme was found in *Pseudomonas fluorescens* and shares 68% amino acid identity with SFC-1 from *Serratia fonticola*, and 59% with plasmid-encoded KPC-2 [61].

There are several ways for CR to enter the environment. These include, for example, wastewater treatment plants (WWTPs), especially those receiving wastewater from hospitals [21,62,63]. WWTPs can release large numbers of ARB into the environment, where they might temporarily survive and even proliferate [7,8,64]. WWTPs typically do not remove antibiotic-resistance genes (ARGs), but instead may spread them into the aquatic environment [8,56,65]. For example, a study in Spain detected clinically relevant ARGs in biofilms and river sediments that were distant to WWTP discharge points [7]. Carbapenemase-producing *Enterobacteriaceae* (CPE), *Acinetobacter* spp., *Aeromonas* spp., and *Pseudomonas* spp. containing the genes *bla*_NDM_, *bla*_KPC,_ and *bla*_OXA_ have also been isolated from raw sewage, treated effluent, and the receiving river waters [56,63]. WWTPs can, therefore, play a significant role in the dissemination of CRB and CRGs.

**Figure 1:**
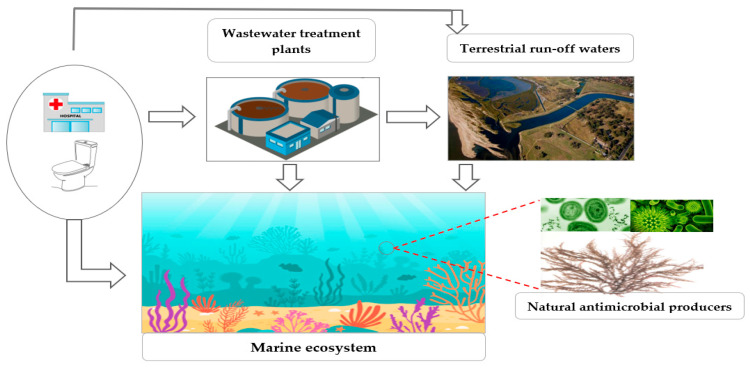
Graphical diagram of the spread of CRB (carbapenem-resistant bacteria) or CRGs (carbapenem-resistant genes) into the marine environment. The diversity of CRB or CRGs in the seawater can be sourced from the human communities and loaded into the marine matrices either via direct sewage disposals or via the outfall of wastewater treatment plants. The marine coastal environment may also receive CR (carbapenem resistance) from polluted rivers and other terrestrial run-off waters. The occurrence of CR in the ocean can be due to the selection pressure of natural β-lactam antibiotics produced by other marine microorganisms.

The further circulation of CR in aquatic matrices may potentially pollute rivers and drinking water sources [66]. A novel class B metallo-β-lactamase was also identified in *Shigella boydii*, *Aeromonas caviae,* and *Vibrio cholerae* from seepage and drinking water samples [67]. Multi-resistant bacteria and ARGs were detected at a drinking water intake at Lake Geneva, Switzerland, which was 3.2 km away from a WWTP outlet [68]. Furthermore, CRB have also been found in drinking water in several parts of the world. For instance, carbapenemase-producing *Serratia fonticola* was reported in drinking water in Portugal [69], and CPE including *E*. *coli*, *Kluyvera*, *Providencia*, *Klebsiella*, and *Citrobacter* species, and non-fermenting Gram-negative species, such as *Shewanella* spp., *Pseudomonas* spp., and *Acinetobacter* spp., were found to contain *bla*OXA-48-type carbapenemase in USA drinking waters [66]. In addition, non-fermenting Gram-negative rods carrying the *bla*_NDM_ gene were isolated from New Delhi drinking water [67]. Thus, the aquatic environments may serve as a vehicle by which CRB or CRGs could be disseminated from one aquatic ecosystem to another (see Figure 1).

The dense bacterial communities in WWTPs can also facilitate genetic exchange between bacteria, which can lead to the horizontal transfer of resistance genes between clinical pathogens and environmental microorganisms or vice versa [7,70]. For example, *Citrobacter freundii* and *Enterobacter cloacae* detected in hospital sewage have been found to contain the same *bla*_KPC-2_ gene [71]. The species *Pseudomonas monteilii*, *Brevundimonas diminuta*, and *Enterobacter ludwigii* have been found to contain the same genetic variant of the *bla*_VIM-13_ gene in sewage [22]. Finding identical CRGs in different bacterial taxa indicates that sewage is a suitable environment for horizontal resistance gene transfer. 

## 4. Distribution of Carbapenem Resistance in Marine Systems

The dissemination of AR in seawater may be influenced by discharges from coastal runoff, aquaculture, polluted rivers, and WWTP effluents, which frequently contain resistant bacteria and resistant genes [72,73,74,75,76,77]. A study described that CRB communities in seawater and storm water samples did not differ significantly at the investigated sites, and the phylogenetic analysis showed that their CR isolates often belonged to the same species [25]. CPE, such as *Enterobacter* spp. and *E*. *coli* carrying *bla*_IMI-2_, were also isolated from river estuaries and beach water. Further molecular analysis and genome comparisons revealed the high similarity of these riverine and marine CRB from samples that were collected one month apart [77]. Thus, local sources, such as stormwaters and rivers, are an important source of CRB in the seawater and may have a significant effect on the composition of CRB in the marine environment (see Figure 1).

Furthermore, CR has also been recently detected in the marine environment [25,73,76,78,79,80]. For example, CRB belonging to a wide range of bacterial taxa, including four phyla, eight classes, and 30 genera, were found in Australian marine and near-shore environments [25]. Many genera found in the marine environment, such as *Pseudomonas* [9,81], *Stenotrophomonas* [81], *Acinetobacter* [51,82,83], *Brevundimonas* [84], *Caulobacter* [85], *Chryseobacterium* [86,87], *Empedobacter* [88], *Sphingomonas* [86], *Flavobacterium* [89], *Cupriavidus* [81], *Myroides* [89], *Ochrobactrum* [90], and *Pedobacter* [86], have already been previously described to contain CR, and these bacteria may carry well-known and clinically relevant CRGs. For example, clinically relevant CRGs, such as KPC-2, Guiana extended spectrum (GES)-like, and OXA-carbapenemases, have been identified in several species, including *Klebsiella* spp., *Citrobacter* spp., *Kluyvera* spp., *Enterobacter cloacae*, *E*. *kobei*, *E*. *asburiae*, *Aeromonas punctata,* and *A*. *hydrophila* isolated from coastal water [73,91]. In addition, NDM genes have also been detected in *K*. *pneumoniae* and *E*. *coli* from beach waters [91,92,93]. Worryingly, CPE, *Acinetobacter* spp., *Aeromonas* spp., and *Pseudomonas* spp. from recreational beach waters have been consistently found throughout the year to carry multiple CRGs, including KPC, GES-like carbapenemase, NDM, IMP, Verona integron-encoded metallo-β-lactamases (VIM), Sao Paulo metallo-β-lactamases (SPM), and OXA-carbapenemase [76] (see Table 1). These studies show that clinically relevant CRGs have entered the marine environment and potentially spread into other bacteria.

## 5. Potential for CR Transfer and Reservoir in the Marine Environment

The marine environment may contribute to the further dissemination of CRGs between different bacterial strains by providing a medium in which horizontal gene transfer can take place [98,99]. For example, a study of CR in the coastal environment found that most of the CR aquatic isolates were assigned to the genus *Pseudomonas*, including the species *P*. *asplenii*, *P*. *monteilii*, *P*. *fulva*, *P*. *plecoglossicida*, *P*. *stutzeri*, *P*. *taiwanensis,* and *P*. *xanthomarina*. In the genus *Pseudomonas* (family *Pseudomonadaceae*), the production of carbapenemases, such as IMP, VIM, NDM, and KPC, is considered to be the predominant mechanism underlying CR [9,18,100,101]. The CRGs are frequently located in the mobile genetic elements, which facilitate their horizontal transfer between different species [102,103,104,105]. Thus, horizontal gene transfer might have been involved in the dissemination of CRGs in the *Pseudomonas* species, similar to what has been observed in the family *Enterobacteriaceae* [40,41,106,107].

Several CRGs, such as *bla*_KPC_, *bla*_NDM_, *bla*_GES_, and *bla*_OXA48-like_, have been found in different members of *Enterobacteriaceae*, including *K*. *pneumoniae*, *Aeromonas punctata*, *A*. *hydrophila*, *E*. *coli*, *E*. *cloacae*, *E*. *kobei,* and *E*. *asburiae*, in recreational seawaters [91,93]. Furthermore, *E*. *coli* from terrestrial sources could transfer its *bla*_CTX-M-15_ gene to *Pantoea agglomerans* and *Raoultella terrigena* in the coastal seawater [108]. Another study reported that in seawater samples, *Rheinheimera* spp., which are typical marine bacteria [109,110], have been found to share an identical MBL gene utilizing plasmid transfer and chromosomal integration with the species *C*. *freundii* and *E*. *cloacae*, which are not typically found in seawater [25]. In addition, *Variovorax* spp. carried the NDM-type genes, which were likely acquired from the species *E*. *coli*, *K*. *pneumoniae*, and *A*. *baumannii.* These utilized plasmids have also been reported in this coastal water study [25].

Marine samples have also been found to house CRB from genera such as *Chromobacterium*, *Rheinheimera*, *Variovorax*, *Aquiflexum*, *Chitinophaga*, *Herbaspirillum*, and *Xanthobacter*, which have not previously been known to have CR, indicating the potential for new resistance genes [25]. As marine environments are not usually exposed to high concentrations of clinically relevant carbapenems or other β-lactam antibiotics [111,112], marine bacteria may evolve novel CR due to the selection pressure of natural β-lactam antibiotics produced by other marine microorganisms [113,114]. This could, for example, include aureoverticillactam and lajollamycin found in the marine species *Streptomyces aureoverticillatus* [115] and *S*. *nodosus* [116], respectively (see Figure 1). As such, several novel carbapenemases have been recently discovered. These include ElBla2 MBL from the species *Erythrobacter litoralis* (family *Sphingomonadaceae*), which has an amino acid sequence similarity to NDM-1 [78]; the PH-1 MBL gene from the species *Pelagibacterium halotolerans* (family *Hyphomicrobiaceae*) [80]; and the RH-B3-MBL gene from the genus *Rheinheimera* (family *Chromatiaceae*) [25]. These findings provide further evidence that the marine environment represents an unexplored reservoir of novel carbapenemases.

## 6. The Effect of Marine CRGs/CRB on Human Health

To date, the impact of pathogenic CRB has been extensively studied within the healthcare setting [33,48,49]. However, several bacteria relevant to human health with CR, including *Pseudomonas fulva*, *Brevundimonas vesicularis*, *Enterococcus durans*, *Acinetobacter junii*, *A*. *johnsonii*, *Microbacterium lacus*, and *S*. *maltophilia*, have been found in coastal seawater in Australia [25]. These species have been found to cause human infections, such as bacteraemia (*Acinetobacter junii*, *A*. *johnsonii*, *Pseudomonas fulva*, and *Brevundimonas vesicularis*) [117,118,119,120], meningitis (*P*. *fulva*) [100], endocarditis (*E*. *durans*) [121], cellulitis, soft tissue, urinary and respiratory tract infections (*Stenotrophomonas maltophilia*, *Microbacterium lacus*) [122,123,124,125], and eye infections (*S*. *maltophilia* and *A*. *junii*) [126,127].

In addition, there is growing evidence that CRGs have been carried by human opportunistic pathogens into seawaters [73,80,94]. For example, CRGs, such as KPC, IMP, VIM, SPM, NDM, and OXA-carbapenemases, have been identified in *E*. *coli*, *K*. *pneumoniae*, *Acinetobacter* spp., *Aeromonas* spp., *Enterobacter* spp., and *Pseudomonas* spp. in recreational coastal waters of Rio de Janeiro [73,76], Israel [77], and Ireland [95].

**Figure 2:**
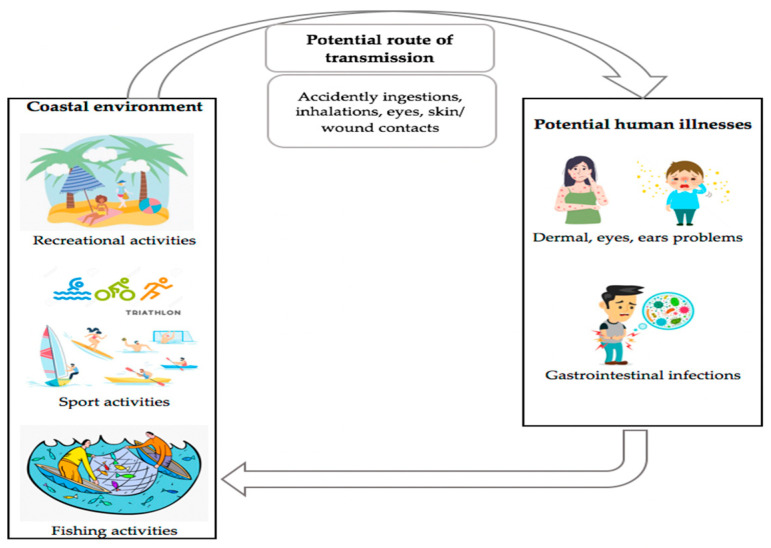
Schematic diagram showing the route of potential CRB transmission from the coastal environment to humans and the potential health problems that may occur. The practice of recreational activities, such as sport and fishing, in CR-contaminated coastal environments may result in potential human illnesses, such as dermal or eye problems and gastrointestinal infections. The possible routes of transmission include accidentally ingested or inhaled beach waters that are contaminated by CR.

This prevalence has public health implications [26,86], since coastal waters are frequently used for recreational and sport purposes. CR infections can, in such circumstances, be acquired through common routes of pathogen exposure to humans, including ingestion, inhalation, and dermal or eye contact with the beach waters [128,129] (see Figure 2). For example, the risk of experiencing symptoms of gastrointestinal, ear, and skin infections from exposure to seawater with bacterial contamination is higher in bathers than in non-bathers [26,129]. Open wounds in soft tissues are also vulnerable to infection by opportunistic human pathogens. For example, a soft tissue infection by *Microbacterium lacus* was acquired after a bather’s elbow was bumped on a rock while swimming [124]. Considering the spread of CRB in marine environments that are frequently used for human activities, the potential risk of CRB transfer from this reservoir to humans is still great. Further in-depth studies to quantify the risk of human infection with these CRB in recreational seawater should be performed.

## 7. Conclusions

The global spread of CR is becoming a major threat to public health and has worsened with the detection of resistance in last-line antibiotics, including carbapenems [1,15,16]. CR has not only been reported in pathogenic bacteria [6,9,10,31,130], but also in environmentally derived bacteria [86,108,131,132]. As non-clinical environments, such as sewage, rivers, lakes, and oceans, may serve as the media for the transmission of CR [24,73,76,133,134], studies of the further dissemination of CR in these matrices are critically important. In the coastal environment, CRB and CRGs seem to be transported from terrestrial sources through stormwater runoff, wastewater discharges, and polluted rivers [21,25,42,57], which had a significant effect on the diversity and CRB load in the marine environment.

To date, a large variety of CRGs have been identified, and the transfer of CRGs between different bacteria has been characterized [22,49,135]. Most CRGs are located on mobile genetic elements, such as plasmids or transposons, and their mobility contributes to the rapid spread of CRGs between bacteria [16,136]. The identification of CRGs, including novel carbapenemases in non-targeted marine bacteria [25,78,80], suggests either that CRGs have been horizontally transferred from non-marine bacteria (e.g., fecal contaminant) to marine bacteria or that novel CRGs have evolved in marine lineages due to continuing selection by naturally produced β-lactam antibiotics in the marine environment.

Given that the CRB identified in coastal environments are relevant to human health, public health concerns may arise due to the fact that beach water is mostly designated for recreational and sporting activities [24,26,73,137]. Future investigation of CR in the coastal environment is necessary to understand the resistant epidemiology and quantify the potential risk for CRB to cause human illnesses.

## Figures and Tables

**Table 1:** Carbapenem-resistant bacteria and carbapenem resistance genes found in coastal environments.

Carbapenem Resistant Bacteria	Carbapenem Resistance Determinants	Reference
*Vibrio cholerae*	Not identified	[24]
*Rheinheimera* spp.	B3-MBL	[25]
*Variovorax* spp.	NDM	
*Enterobacteriaceae*	KPC, OXA	[72]
*Citrobacter* sp., *Citrobacter* sp., *Kluyvera* sp., *Aeromonas* sp.	KPC-2	[73]
*Acinetobacter* spp.	OXA	[76]
*Aeromonas* spp.	KPC-2, GES-5, GES-16	
*Citrobacter* sp.	KPC-2, OXA-370	
*Enterobacter* spp.	KPC-2, KPC-26, GES-5, GES-16	
*Klebsiella* spp.	KPC-2, KPC-26, GES-16, NDM-1	
*Kluyvera* spp., *Serratia* spp.	KPC-2	
*Pseudomonas* spp.	VIM-2, SPM-1	
*Enterobacter asburiae*	IMI-2	[77]
*Enterobacter bugandensis*	IMI-20	
*Escherichia coli*	OXA-48	
*Erythrobacter litoralis*	ElBla2 *	[78]
*Enterobacter cloacae*	KPC-2, CTX-M-15, OXA-17	[79]
*Pelagibacterium halotolerans*	PH-1 *	[80]
*Aeromonas punctata*, *Enterobacter asburiae*, *K*. *pneumoniae*, *Enterobacter kobei*	KPC, GES-16, OXA-48-like	[91]
*K*. *pneumoniae*	NDM	[93]
*K*. *pneumoniae*	NDM-1, OXA-1	[92]
*Pseudomonas* spp., *Rheinheimera* spp., *Stenotrophomonas* sp., *Shewanella* sp., *Raoultella* sp., *Vibrio* sp., *Pseudoalteromonas* sp., *Algoriphagus* sp., *Bowmanella* sp., *and Thalassospira* sp.	OXA-58	[94]
*E*. *coli*, *K*. *pneumoniae*	OXA-48	[95]
*Shewanella livingstonensis*	SLB-1 *	[96]
*Shewanella frigidimarina*	SFB-1 *	
*Aliivibrio salmonicida*	ALI-1 *	[97]

* Novel metallo-β-lactamase.

## Data Availability

No new data were created or analyzed in this study. Data sharing is not applicable to this article.
